# Graphene Oxide Quantum Dots Promote Osteogenic Differentiation of Stem Cells from Human Exfoliated Deciduous Teeth via the Wnt/*β*-Catenin Signaling Pathway

**DOI:** 10.1155/2021/8876745

**Published:** 2021-02-05

**Authors:** Xin Yang, Qi Zhao, JingWen Chen, Jiayue Liu, Jiacheng Lin, Jiaxuan Lu, Wenqing Li, Dongsheng Yu, Wei Zhao

**Affiliations:** ^1^Guanghua School of Stomatology, Hospital of Stomatology, Guangdong Provincial Key Laboratory of Stomatology, Sun Yat-sen University, Guangzhou 510055, China; ^2^Xianning Central Hospital, The First Affiliated Hospital of Hubei University of Science And Technology, Xianning 437000, China

## Abstract

Graphene oxide quantum dots (GOQDs) are a carbon nanomaterial with broad potential for application in the field of nanomaterial biomedicine. Stem cells from human exfoliated deciduous teeth (SHEDs) play an important role in tissue engineering and regenerative medicine. This study investigated the effects of GOQDs on SHED osteogenic differentiation. GOQDs were synthesized; then, the proliferation of SHEDs incubated in GOQDs at different concentrations was evaluated; and the live cells were observed. We observed that live SHEDs incubated in GOQDs emitted green fluorescence in the absence of chemical dyes, and 1, 10, and 50 *μ*g/mL GOQDs significantly promoted SHED proliferation. Culture with the osteogenic induction medium containing 10 *μ*g/mL GOQDs induced calcium nodule formation, increased alkaline phosphatase (ALP) activity, and upregulated SHED mRNA and protein levels of OCN, RUNX2, COL I, and *β*-catenin. With the addition of Dickkopf 1 (DKK-1) or *β*-*catenin* knockdown, expression levels of the above mRNAs and proteins were decreased in GOQD-treated SHEDs. In summary, at a concentration of 10 *μ*g/mL, GOQDs promote SHED proliferation and osteogenic differentiation via the Wnt/*β*-catenin signaling pathway. This work provides new ideas and fundamental information on interactions between GOQDs and SHEDs that are relevant for the biomedical engineering field.

## 1. Introduction

Inflammation, trauma, and tumors can cause bone tissue defects that seriously affect the patient's physical and mental health. A growing number of recent studies have focused on bone tissue engineering technologies to address these issues. This refers to the technique of bone regeneration and repair of bone defects by inducing osteogenic differentiation in stem cells, accurately forming the desired tissue for the defect *in vitro*, and then transplanting it back into the patient [1–4]. It is vital to continuously develop improved materials to induce osteogenic differentiation in stem cells and demonstrate the mechanisms.

In 2003, Miura and colleagues isolated a type of cell from the pulp of deciduous teeth that is highly self-renewable and has extensive proliferation and multidirectional differentiation potential that they called SHEDs [5]. There are many convenient ways to obtain SHEDs that do not cause trauma, and they carry a low risk of immune rejection or cross infection [6–8]. SHEDs can be obtained from fresh autologous tissue and used after cryopreservation; there is no obvious difference between the fresh and cryogenic SHEDs in their abilities to proliferate and undergo multidirectional differentiation [9]. This approach allows the establishment of a human pulp stem cell bank and provides a resource for stem cell therapy. SHEDs also have potent secretion and immune regulation properties [10, 11] and have been shown in animal models to have potential therapeutic effects for bone defects, diabetes, and autoimmune, neurodegenerative, liver, and kidney diseases. Some of these research results have been applied in clinical practice [10–12]. SHEDs are already playing an important role in the fields of tissue engineering and regenerative medicine. In a previous study, our group successfully separated and cultured SHEDs and demonstrated that 1 *μ*g/mL GOQDs promoted SHED proliferation [13].

GOQDs are a carbon nanomaterial of several nanometers per dot and have important properties of quantum confinement effect, stable photoluminescence (PL), and good biocompatibility [14]. For these reasons, they have attracted much attention from scientists and exhibit promise for application in many fields, including bioimaging devices [15], drug delivery [16], electrochemical biosensors [17], fuel cells [18], and other useful products. Sun et al. [19] demonstrated that GOQDs can be used as fluorescence probes in fluorescence labeling fields and live cell imaging due to their excellent PL. Wang and colleagues [20] showed that GOQDs can be used to deliver genes, proteins, and drugs due to their ability to be structured over a large surface area. Choi et al. [21] reported that GOQDs can be loaded with medicine and used as a nontoxic light-sensitive agent in photodynamic therapy to treat clinical tumors. In addition, our group demonstrated that GOQDs at certain concentrations can induce osteogenic differentiation in stem cells, which supports their application in tissue regeneration [13]. In that work, we speculated that GOQDs may induce osteogenic differentiation in SHEDs via the Wnt/*β*-catenin signaling pathway, which called for further investigation.

The Wnt/*β*-catenin signaling pathway is a highly evolutionarily conserved pathway involved in regulating multiple biological processes [22]. The process of accumulating *β*-catenin in the cytoplasm and its subsequent translocation to the nucleus are important signs of pathway activation, which leads to the transcription of proteins in the Wnt/*β*-catenin pathway and downstream activation of target genes [23]. Many recent studies have focused on the role of Wnt/*β*-catenin signaling in bone tissue, and it has been shown to regulate osteogenic differentiation in human dental pulp stem cells (hDPSCs). Jiang et al. [24] found that after *kif3a* in hDPSCs was knocked down and Wnt3a was added to the osteogenic induction medium, the expression of the osteoblastic marker ALP and the Wnt/*β*-catenin signaling pathway marker *β*-catenin were significantly increased. This suggested that *kif3a* might promote osteogenic differentiation of hDPSCs. Li et al. [25] demonstrated that a high concentration of basic fibroblast growth factor (bFGF) could inhibit *β*-catenin expression, thus inhibiting SHED osteogenic differentiation. Liu et al. [26] indicated that *β*-catenin can act as a downstream regulatory protein for telomerase reverse transcriptase. They found that low-concentration acetylsalicylic acid upregulated Wnt/*β*-catenin pathway protein expression by improving the activity of telomerase reverse transcriptase and enhancing *β*-catenin expression, ultimately promoting SHED osteogenic differentiation. Our group has found that the expression trend for *β*-catenin following GOQD treatment is the same as that for osteoblastic markers, which induce osteogenic differentiation in SHEDs [13].

In the present study, we investigated the effects of GOQDs on SHED osteogenic differentiation and explored the underlying mechanism. First, we prepared GOQDs using a bottom-up method [27]; then, we evaluated the proliferation of SHEDs incubated with GOQDs at different concentrations, observing live cells with confocal laser scanning microscopy. We then quantified calcium nodules, ALP activity, and mRNA and protein levels related to osteogenic differentiation and the Wnt/*β*-catenin signaling pathway. DKK-1 was used to inhibit the Wnt/*β*-catenin signaling pathway, and *β*-*catenin* was knocked down to verify the mechanism by which GOQDs induce SHED osteogenic differentiation. We hypothesized that at a concentration of 10 *μ*g/mL, GOQDs would promote osteogenic differentiation via the Wnt/*β*-catenin signaling pathway.

## 2. Materials and Methods

### 2.1. GOQD Preparation and Characterization

GOQDs were prepared by directly pyrolyzing citric acid (CA; Macklin, Shanghai, China) using the bottom-up method described previously [27]. In detail, 2.5 g CA and 25 mL double-distilled water (ddH_2_O) were put into a beaker and heated to 200°C for 2–3 h. During the heating process, the CA was dissolved, and the colorless liquid changed to yellow and finally to orange, indicating GOQD formation. Next, the orange liquid was dialyzed in a 500 Da molecular weight dialysis bag for 48 h to remove CA, and then the liquid was freeze-dried to yield solid GOQDs, which were dissolved in tri-distilled water to obtain a GOQD suspension at a concentration of 1 mg/mL and sterilized with a 0.22 *μ*m filter membrane (EMD Millipore, Billerica, MA, USA).

The nanosize morphologies of the GOQDs were characterized by transmission electron microscopy (TEM; FEI Tecnai G2 Spirit, Hillsboro, OR, USA) at an acceleration voltage of 300 kV. The compositions of GOQDs were characterized by Fourier transform infrared spectrometry (FTIR; Bruker, Karlsruhe, Germany). The ultraviolet- (UV-) visible spectra of GOQDs were measured on a Lambda 950 spectrophotometer (PerkinElmer, Waltham, MA, USA). The fluorescence spectra of GOQDs under 365 nm excitation wavelengths were measured on a Cary Eclipse (Agilent Technologies, Santa Clara, CA, USA).

### 2.2. Proliferation Assay

Ethics committee approval was provided by the School of Stomatology, Sun Yat-sen University. SHEDs were isolated and identified as described previously [13]. The cytocompatibility of different concentrations of GOQDs was evaluated with a Cell Counting Kit-8 (CCK-8; Beyotime Institute of Biotechnology, Haimen, China). SHEDs were cultured in a culture medium, including Dulbecco's modified Eagle's medium (DMEM; Gibco, Thermo Fisher Scientific, Inc., Waltham, MA, USA) with 10% fetal bovine serum (FBS; Gibco) and 1% penicillin/streptomycin (P/S; Gibco) at a concentration of 5 × 10^3^ cells/well on 96-well plates, and then the SHEDs were treated with GOQDs at concentrations of 1, 10, 50, 100, and 200 *μ*g/mL. After 1, 3, 5, and 7 days, the culture medium was replaced with 10 *μ*L CCK-8 solution and 100 *μ*L DMEM, and the plates were incubated at 37°C for 1 h. The optical density (OD) at 450 nm was measured using a microplate reader (Tecan, Männedorf, Switzerland).

### 2.3. Live Cell Imaging

The morphology of SHEDs treated with GOQDs was observed via confocal laser scanning microscopy (Zeiss, Oberkochen, Germany). SHEDs were plated on Petri dishes at a concentration of 1 × 10^4^ cells/dish, then treated with GOQDs at concentrations of 1, 10, and 50 *μ*g/mL. After 3 days, SHEDs were washed with phosphate-buffered saline (PBS; Hyclone, Logan, UT, USA) and imaged.

### 2.4. Alizarin Red Staining

SHEDs were seeded in six-well plates; upon reaching 70–80% confluence, they were treated with the osteogenic induction medium (OIM) created from DMEM with 10% FBS, 1% P/S, 1 *μ*M dexamethasone (Sigma-Aldrich, St. Louis, MO, USA), 10 mM *β*-glycerophosphate (Sigma-Aldrich), and 50 *μ*M ascorbic acid (Sigma-Aldrich), with 1, 10, or 50 *μ*g/mL GOQDs, for 14 days. After culturing, the cells were fixed in 4% paraformaldehyde for 30 min, rinsed with PBS, stained with alizarin red (Cyagen Biosciences Inc., Guangzhou, China) for 30 min at room temperature, and washed with PBS. Finally, mineralized nodules were photographed with an inverted microscope (Axiovert 40; Zeiss). The control group was incubated in OIM without GOQDs.

### 2.5. ALP Activity Assay

SHEDs were seeded in 24-well plates, and when 70–80% confluence was reached, they were cultured in OIM with 1, 10, or 50 *μ*g/mL GOQDs for 3 and 7 days. The control group was incubated with OIM without GOQDs. ALP activity was determined using an ALP assay kit (Nanjing Jiancheng Bioengineering Institute, Nanjing, China), according to the manufacturer's instructions.

### 2.6. Quantitative Real-Time Reverse Transcription Polymerase Chain Reaction (qRT-PCR)

SHEDs were seeded in six-well plates, and when they reached 70–80% confluence, they were cultured in OIM, with 1, 10, or 50 *μ*g/mL GOQDs for 7 and 14 days. The control group was incubated in OIM without GOQDs. Total RNA was extracted using the TRIzol reagent (Invitrogen, Carlsbad, CA, USA), following the manufacturer's protocol. PrimeScript™ RT Master Mix (Takara Bio Inc., Shiga, Japan) was used to convert messenger RNA (mRNA) into complementary DNA (cDNA). Then, the cDNA was quantified using qRT-PCR, which was performed on a LightCycler 480 Detection System (Roche, Basel, Switzerland) with an SYBR Green Kit (Roche) using gene-specific primers. The primer sequences are shown in Table 1. Glyceraldehyde 3-phosphate dehydrogenase (GAPDH) was used as an internal control.

### 2.7. Western Blot (WB) Assay

SHEDs were seeded in six-well plates, and when they reached 70–80% confluence, they were cultured in OIM with 1, 10, or 50 *μ*g/mL GOQDs for 14 days. The control group was incubated in OIM without GOQDs. Briefly, the radioimmunoprecipitation assay buffer (KeyGen BioTECH, Nanjing, China) containing 1 mmol/L protease inhibitor cocktail (CWBIO, Beijing, China) was used to lyse cells, a bicinchoninic acid (BCA) assay kit (CWBIO) was used to measure protein concentrations, and sodium dodecyl sulfate-polyacrylamide gel electrophoresis (SDS-PAGE, CWBIO) was used to separate proteins that were then transferred onto polyvinylidene fluoride membranes (Millipore). Next, 5% fat-free milk in Tris-buffered saline with Tween (TBST; 10 mmol/L Tris-HCl, 50 mmol/L NaCl, and 0.25% Tween 20) was used to block membranes for 1 h at room temperature, which were then incubated with primary antibodies (anti-osteocalcin (OCN) and anti-collagen I (COL I), 1 : 500, Abcam, Cambridge, UK; anti-runt-related transcription factor-2 (RUNX2), anti-*β*-catenin, and anti-GAPDH, 1 : 1000, Cell Signaling Technology (CST), Danvers, MA, USA) for 18 h at 4°C. Finally, the membranes were incubated with horseradish peroxidase-conjugated anti-rabbit secondary antibodies (1 : 2000, CST), and bands were detected with an enhanced chemiluminescence detection system (Millipore) and quantified using ImageJ v1.47 software (National Institutes of Health, Bethesda, MD, USA). GAPDH was used as an internal control.

### 2.8. Effects of DKK-1

After the above steps, SHEDs were treated with GOQDs at a concentration of 10 *μ*g/mL to induce osteogenic differentiation for the next experiment. SHEDs were seeded in six-well plates, and when they reached 70–80% confluence, they were cultured in OIM with GOQDs and with or without 100 ng/mL DKK-1. The cells were divided into three groups: control (SHEDs cultured in OIM alone), DKK-1 (SHEDs cultured in OIM, DKK-1, and GOQDs), and GOQD (SHEDs cultured in OIM and GOQDs). The effects of DKK-1 relating to the GOQD-mediated induction of osteogenic differentiation in SHEDs were confirmed via qRT-PCR and WB assays.

### 2.9. Lentiviral Infection

SHEDs were seeded into a culture flask, and when they reached 50% confluence, they were treated with DMEM containing enhanced green fluorescence protein-labeled (EGFP) *β*-catenin small interfering RNA (siRNA) lentivirus (Shanghai Genechem Co., Ltd., Shanghai, China). siRNA against *β*-catenin GTATTTGAAGTATACCATA and a nonspecific shRNA construct were designed and cloned onto an hU6-MCS-CMV-EGFP vector. Control viruses that contained the EGFP tag (Shanghai Genechem Co., Ltd.) were also provided. After incubation for 10 h at 37°C and 5% CO_2_, the supernatant was removed and replaced with a culture medium for 3 days. Infected cells were imaged with a fluorescence inverted microscope (Zeiss), and the knockdown effect was confirmed via qRT-PCR and WB assays. SHEDs infected with *β*-catenin recombinant lentiviruses or empty lentiviral vectors were defined as the *β*-catenin-KD group and the control-si group, respectively.

### 2.10. Effects of *β*-*Catenin*

SHEDs were treated with 10 *μ*g/mL GOQDs to induce osteogenic differentiation for the next experiment. They were seeded in six-well plates, and upon reaching 70–80% confluence, they were cultured in OIM that included GOQDs. The cells were independently divided into three groups: control-si (SHEDs infected with control viruses and cultured in OIM alone), GOQD-si (SHEDs infected with control viruses and cultured in OIM and GOQDs), and *β*-catenin-si (SHEDs infected with *β*-catenin recombinant lentiviruses and cultured in OIM and GOQDs). The effects of *β*-*catenin* on GOQD-mediated induction of osteogenic differentiation in SHEDs were confirmed via qRT-PCR and WB assays.

### 2.11. Statistical Analysis

The data are shown as means ± standard deviations. One-way analysis of variance was used to perform statistical analyses, and the Bonferroni method was used for multiple comparisons. Differences were considered significant at *p* < 0.05. SPSS 20.0 software (SPSS Inc., Chicago, Illinois, USA) was used for all statistical analyses.

## 3. Results

### 3.1. Synthesized GOQDs

**Figure 1:**
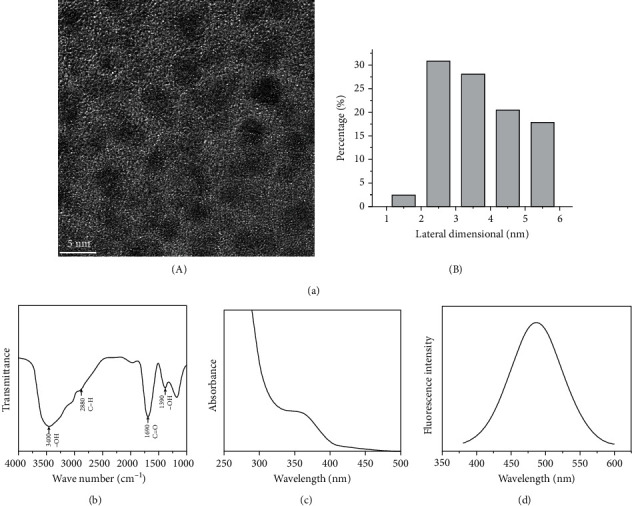
Characterization of GOQDs. (a) (A) TEM image of GOQDs. (B) Lateral size distribution of GOQDs from (A). (b) FTIR spectroscopy of GOQDs. (c) UV-visible spectrum of GOQDs. (d) PL spectrum of GOQDs excited at a wavelength of 365 nm.

GOQD morphology was observed using TEM (Figure 1(a)); they were dispersed and had dot sizes with diameters largely under 10 nm. Surface chemistry was investigated by FTIR (Figure 1(b)), and absorption bands at 3400 cm^−1^ and 1390 cm^−1^ were observed due to stretching in hydroxyl groups (–OH) and stretching vibrations in carboxyl groups (C=O) at 1690 cm^−1^ and hydrocarbon groups (C–H) at 2880 cm^−1^. As shown in Figures 1(c) and 1(d), the excitation wavelength of GOQDs is 365 nm, and the emission spectrum ranges from 380 to 600 nm, indicating that GOQDs have PL.

### 3.2. GOQDs Promote SHED Proliferation

**Figure 2:**
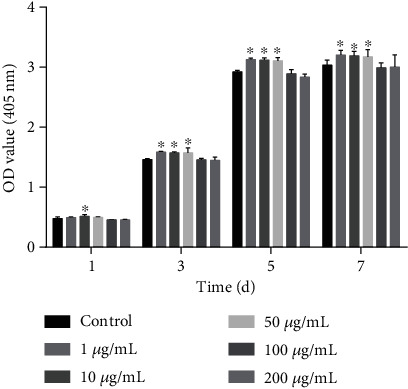
The proliferation of SHEDs incubated with different concentrations (0, 1, 10, 50, 100, and 200 *μ*g/mL) of GOQDs for 1, 3, 5, and 7 days. ^∗^*p* < 0.05 vs. the control group.

CCK-8 assays were used to evaluate SHED viability (Figure 2). The results showed that 1, 10, and 50 *μ*g/mL GOQDs significantly promoted SHED proliferation at days 3, 5, and 7. At the highest concentration of 200 *μ*g/mL, GOQDs slightly suppressed cell viability (but without a statistical difference). Therefore, we chose the 1, 10, and 50 *μ*g/mL concentrations of GOQDs to perform the following assays.

### 3.3. SHEDs Incubated in GOQDs Emitted Green Fluorescence

**Figure 3:**
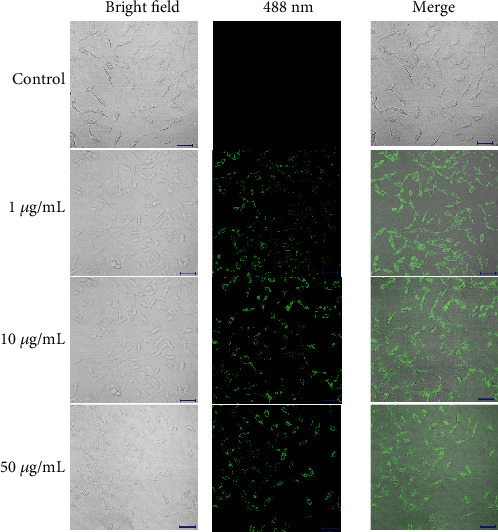
Confocal laser scanning microscopy images of SHEDs incubated with 1, 10, and 50 *μ*g/mL of GOQDs, respectively, for 3 days. Scale bar, 100 *μ*m.

*In vitro* cell images are shown in Figure 3. After incubation with GOQDs at concentrations of 1, 10, and 50 *μ*g/mL, SHEDs emitted green fluorescence, predominantly in the cytoplasm, under illumination at a wavelength of 488 nm. SHEDs that were not exposed to GOQDs and imaged under the same conditions did not show any fluorescence. In all groups, SHEDs showed a normal spindle-like or fibroblast-like morphology.

### 3.4. Mineralized Nodule Formation

**Figure 4:**
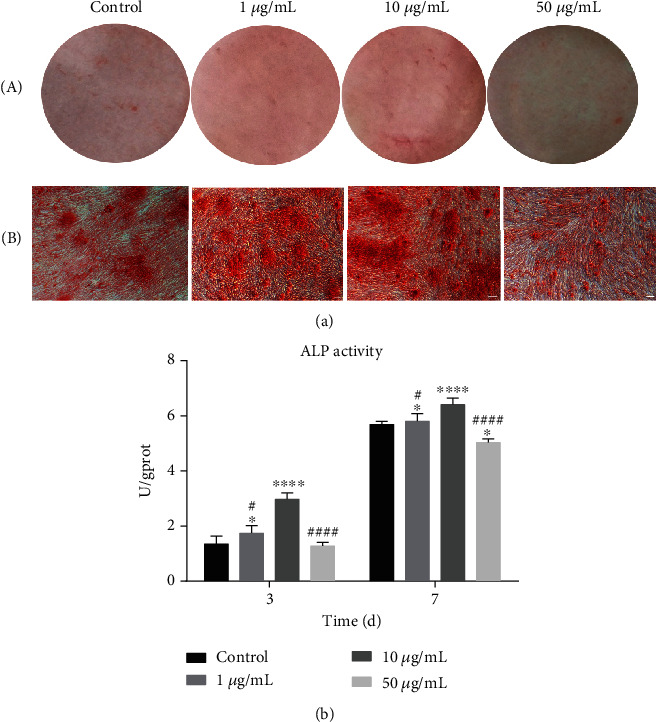
SHEDs were cultured in the osteogenic induction medium containing GOQDs. Osteogenic differentiation was detected by (a) alizarin red S staining for 14 days: (A) optical photos and (B) microscopic images. (b) ALP activity after 3 and 7 days. Scale bar, 100 *μ*m. ^∗^*p* < 0.05, ^∗∗^*p* < 0.01, ^∗∗∗^*p* < 0.001, and ^∗∗∗∗^*p* < 0.0001 vs. the control group. ^#^*p* < 0.05, ^##^*p* < 0.01, ^###^*p <* 0.001, and ^####^*p* < 0.0001 vs. the 10 *μ*g/mL GOQD group.

Calcium nodules were formed in all four groups after osteogenic induction for 14 days (Figure 4(a)). Calcium nodule formation in the 1 and 10 *μ*g/mL GOQD groups was greater than that in the control group, and it was less evident in the 50 *μ*g/mL GOQD group. Calcium nodule formation in the 1 *μ*g/mL group was greater than that in the 50 *μ*g/mL group but less than that in the 10 *μ*g/mL group. The highest level of mineralization was observed in the 10 *μ*g/mL group.

### 3.5. GOQDs Increased ALP Activity in SHEDs

After osteogenic differentiation of SHEDs for 3 and 7 days (Figure 4(b)), ALP activity in the 10 *μ*g/mL GOQD group was significantly higher than that in the other groups. ALP activity was increased in the 1 and 10 *μ*g/mL GOQD groups compared to the control group, and it was significantly decreased in the 50 *μ*g/mL GOQD group.

### 3.6. GOQDs Upregulated SHED mRNA Levels

**Figure 5:**
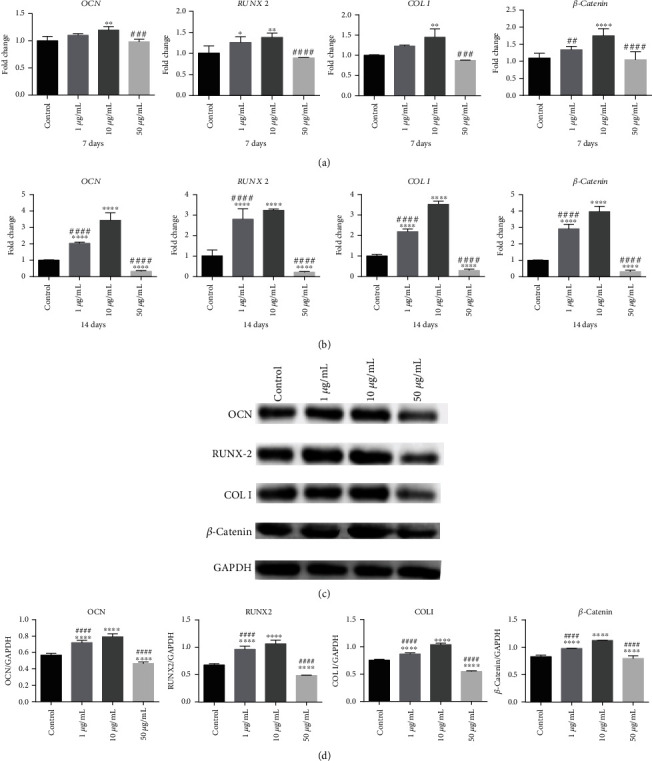
SHEDs were cultured in the osteogenic induction medium containing GOQDs. (a) mRNA extracted at 7 days. (b) mRNA extracted at 14 days. (c) Protein extracted at 14 days. (d) Quantification of protein levels. ^∗^*p* < 0.05, ^∗∗^*p* < 0.01, ^∗∗∗^*p* < 0.001, and ^∗∗∗∗^*p* < 0.0001 vs. the control group. ^#^*p* < 0.05, ^##^*p* < 0.01, ^###^*p* < 0.001, and ^####^*p* < 0.0001 vs. the 10 *μ*g/mL GOQD group.

The qRT-RCR results following GOQD-induced osteogenic differentiation of SHEDs for 7 days are shown in Figure 5(a). The expression levels of the osteogenic mRNAs *OCN*, *RUNX2*, and *COL I* and the Wnt/*β*-catenin signaling pathway-related mRNA *β*-*catenin* were higher in the 1 and 10 *μ*g/mL GOQD groups and lower in the 50 *μ*g/mL GOQD group. After 14 days of induction (Figure 5(b)), mRNA expression in the 10 *μ*g/mL GOQD group was significantly higher than that in the other groups.

### 3.7. GOQDs Upregulated SHED Protein Levels

Protein levels of OCN, RUNX2, COL I, and *β*-catenin were analyzed after 14 days of osteogenic-inducing culture. As shown in Figures 5(c) and 5(d), expression was distinctly higher in the 1 and 10 *μ*g/mL groups and distinctly lower in the 50 *μ*g/mL group compared to the control group. Moreover, it was greater in the 10 *μ*g/mL group than in the 1 *μ*g/mL group.

### 3.8. DKK-1 Suppresses Osteoblastic Differentiation of GOQD-Treated SHEDs

**Figure 6:**
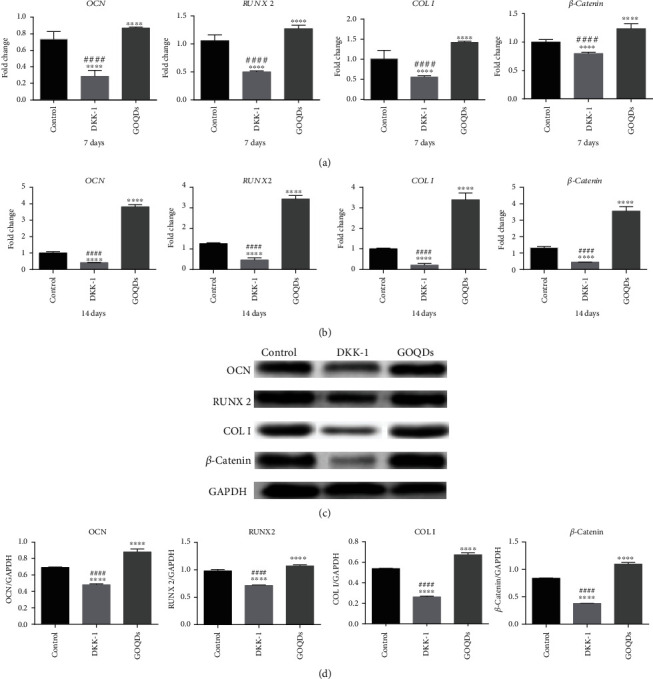
SHEDs were cultured in the osteogenic induction medium containing GOQDs and/without DKK-1. (a) mRNA extracted at 7 days. (b) mRNA extracted at 14 days. (c) Protein extracted at 14 days. (d) Quantification of protein levels. ^∗^*p* < 0.05, ^∗∗^*p* < 0.01, ^∗∗∗^*p* < 0.001, and ^∗∗∗∗^*p* < 0.0001 vs. the control group. ^#^*p* < 0.05, ^##^*p* < 0.01, ^###^*p* < 0.001, and ^####^*p* < 0.0001 vs. the GOQD group.

As shown in Figure 6, OCN, RUNX2, COL I, and *β*-catenin mRNA and protein levels were significantly lower in the control and DKK-1 groups compared to the GOQD group. The lowest level was observed in the DKK-1 group.

### 3.9. Knockdown of *β*-*Catenin* Suppresses Osteoblastic Differentiation in GOQD-Treated SHEDs

**Figure 7:**
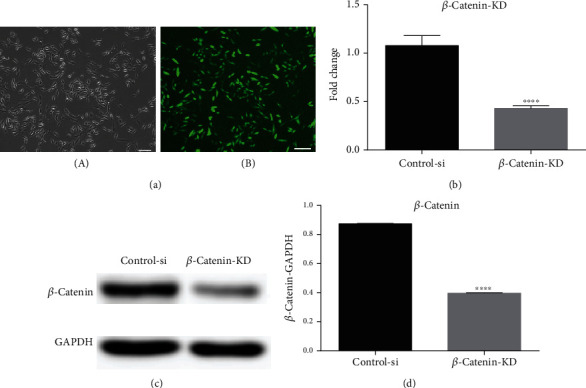
(a) A green fluorescence protein marker was used to determine the transfer efficiency of *β*-*catenin* knockdown in SHEDs (SHED-si). After transfection for 72 h, cells were observed under a (A) contrast phase microscope and an (B) immunofluorescence microscope. Scale bar, 100 *μ*m. (b) qRT-PCR analysis was performed to detect *β*-*catenin* expression. (c) WB was conducted to determine *β*-catenin. (d) Quantification of *β*-catenin protein levels. ^∗^*p* < 0.05 vs. the control-si group.

Lentiviral gene transfer efficiency in SHEDs was high after 72 h transfection (Figure 7(a)). At the mRNA and protein levels, *β*-catenin was approximately 50% lower in the *β*-catenin-KD group than in the control-si group, suggesting that *β*-*catenin* was effectively silenced (Figures 7(b)–7(d)).

**Figure 8:**
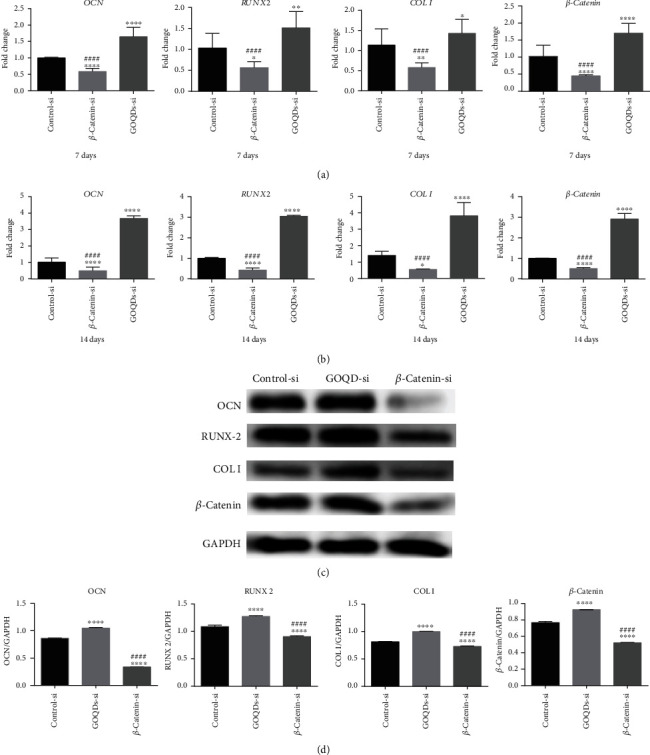
SHEDs were cultured in the osteogenic induction medium containing GOQDs. (a) mRNA extracted at 7 days. (b) mRNA extracted at 14 days. (c) Protein extracted at 14 days. (d) Quantification of protein levels. ^∗^*p* < 0.05, ^∗∗^*p* < 0.01, ^∗∗∗^*p* < 0.001, and ^∗∗∗∗^*p* < 0.0001 vs. the control-si group. ^#^*p* < 0.05, ^##^*p* < 0.01, ^###^*p* < 0.001, and ^####^*p* < 0.0001 vs. the *β*-catenin-si group.

To investigate the differentiation potential of SHEDs after *β*-*catenin* knockdown, we measured mRNA and protein levels of OCN, RUNX2, COL I, and *β*-catenin. After the induction of osteogenic differentiation in SHEDs, *β*-*catenin* knockdown resulted in lower values for the above mRNAs and proteins in the *β*-catenin-si group compared to the control-si and GOQD-si groups (Figure 8).

## 4. Discussion

GOQD synthesis can be divided into two methods: top-down and bottom-up [14, 27]. In the top-down method, graphene or graphite-related materials are cut to the size of a quantum dot using physical, chemical, or electrochemical methods [14]. The bottom-up method uses specific molecular precursors that undergo a gradual chemical reaction to synthesize GOQDs [14]. In this study, we prepared GOQDs using a bottom-up method by pyrolyzing CA [27].  Figure 1 presents a characterization of the GOQDs in this study, showing that they generally had diameters under 10 nm and presented hydrophilicity and PL. In contrast to organic quantum dots, inorganic quantum dots, and fluorescent agents, the most advantageous properties of the GOQDs in this study were their stable PL, good hydrophilicity, and low cytotoxicity [28].

A cytotoxicity assessment was conducted to determine the appropriate concentration of GOQDs to induce SHED proliferation. Cell growth curves are often used to measure logarithmic growth, which reflects the effects of materials on cell viability [29]. A cell growth curve consists of four stages: plateau, logarithmic growth, second plateau, and degenerate aging [29]. As seen in Figure 2, SHEDs demonstrated a strong self-renewal ability and could grow and proliferate in a culture medium that contained a suitable amount of GOQDs. Therefore, GOQD concentrations of 1, 10, and 50 *μ*g/mL GOQDs were used in subsequent experiments. This matched the concentrations of graphene quantum dots (GQDs) used by Qiu et al. [30]. Their group found that GQDs at the concentrations of 1, 10, and 50 *μ*g/mL did not significantly inhibit mesenchymal stem cell (MSC) proliferation.

Many researchers are currently employing GOQDs in cell imaging [19, 28], real-time molecular tracking of living cells [31], and *in vivo* optical imaging [32]. Zhang et al. [28] used GQDs in cell imaging and found that they could enter stem cells easily, had low cytotoxicity, and produced clear and stable stem cell images. Kim et al. [33] demonstrated that GQDs could be used as biological imaging probes to track human adipose-derived stem cells due to their good biocompatibility and highly sensitive optical properties. In this study, we found that GOQDs could be used as a live cell imaging agent. SHEDs emitted green fluorescence and maintained normal stem cell morphology after incubation with GOQDs (Figure 3).

Next, the effects of GOQD exposure on the osteogenic differentiation of SHEDs were evaluated. Mineralized nodules were significantly increased in the 10 *μ*g/mL GOQD group (Figure 4(a)), indicating that this might be a suitable concentration for inducing SHED osteogenic differentiation. However, GOQDs helped promote SHED proliferation, resulting in an increased number of cells. It was therefore necessary to more precisely determine whether the increased number of mineralized nodules in the GOQD group was due to more cells or the tendency of GOQDs to promote osteogenic differentiation. ALP activity assays were then performed. ALP is synthesized by osteoblasts during the early stage of osteogenic differentiation. Its activity reflects the ability of cells to mineralize [34, 35]. Figure 4(b) shows that GOQDs at a concentration of 10 *μ*g/mL had a stronger ability to induce SHED osteogenic differentiation in the early stage. The increase in ALP activity was concentration-dependent.

To investigate further, qRT-PCR and WB assays were used to measure the mRNA and protein expression of osteogenic-related markers. OCN is a matrix protein expressed in the early stages of mineralization and highly expressed in the late stages of osteoblastic differentiation, and it plays an important role in bone tissue mineralization [36]. RUNX2 is an essential transcription factor expressed in the late stages of mineralization that plays a central role in the signal transduction pathway during osteogenic differentiation [37]. COL I is a type of collagen that provides a structure for mineralization; it is an important early expressed protein in osteogenic differentiation [38].  Figure 5 shows the qRT-PCR and WB results, which were consistent with those of alizarin red staining and ALP activity assays. At a concentration of 10 *μ*g/mL, GOQDs had a stronger ability to induce osteogenic differentiation in SHEDs, while it was slightly inhibited at 50 *μ*g/mL. However, Qiu et al. [30] found that 50 *μ*g/mL GQDs had a better ability to induce MSC osteogenic differentiation, while 10 *μ*g/mL GQDs had a greater ability to induce lipogenic differentiation in MSCs. The different physical and chemical properties of GOQDs and GQDs may be the reason for this discrepancy. In addition, Geng et al. [39] found that 10 *μ*g/mL nitrogen-doped GQDs (N-GQDs) were better able to induce osteogenic differentiation in bone MSCs than 50 *μ*g/mL N-GQDs, matching the optimal concentration of GOQDs found in this study.

The mRNA and protein expression trends of the Wnt/*β*-catenin signaling pathway-related factor, *β*-catenin, were in accordance with those of OCN, RUNX2, and COL I. Upregulated *β*-catenin and its nuclear translocation are considered markers of Wnt/*β*-catenin pathway activation [22, 23]. Therefore, we speculated that the Wnt/*β*-catenin signaling pathway might regulate the GOQD-mediated induction of osteogenic differentiation in SHEDs. DKK-1 is an inhibitor of the Wnt/*β*-catenin signaling pathway [40], which can bind to the low-density lipoprotein receptor-related proteins 5 and 6 and then prevent their combination with Frizzled, thus inhibiting accumulation of *β*-catenin in the cytoplasm and blocking signal transduction of the Wnt/*β*-catenin signaling pathway [41, 42]. In this study, we added DKK-1 to the 10 *μ*g/mL GOQD group to verify whether this could antagonize GOQD-induced osteogenic differentiation in SHEDs and preliminarily explored how the Wnt/*β*-catenin signaling pathway regulates this process. The results are shown in Figure 6. DKK-1 inhibited the signaling pathway and reduced *β*-catenin expression, thus antagonizing the ability of GOQDs to induce osteogenic differentiation in SHEDs. To confirm this, *β*-*catenin* was silenced (Figure 7), and the results of the effect of *β*-*catenin* knockdown on SHED osteogenic differentiation are shown in Figure 8. *β*-*Catenin* knockdown reduced OCN, RUNX2, and COL I at the mRNA and protein levels, which supports our assessment of the effects of DKK-1.

Finally, the *in vitro* results demonstrated that GOQDs promote SHED proliferation and osteogenic differentiation in a concentration-dependent manner and that the Wnt/*β*-catenin signaling pathway is activated during this process. However, further *in vivo* research is necessary to verify these results.

## 5. Conclusion

GOQDs promote concentration-dependent proliferation and osteogenic differentiation of SHEDs, and the Wnt/*β*-catenin signaling pathway is involved in regulating these effects. This work provides new ideas and fundamental information on the interactions between GOQDs and SHEDs that are relevant for the biomedical engineering field.

## Figures and Tables

**Table 1:** Primer sequences used in qRT-PCR.

Gene	Primer	Sequence
*OCN*	Forward Reverse	5′-AGCAAAGGTGCAGCCTTTGT-3′ 5′-GCGCCTGGGTCTCTTCACT-3′
*RUNX2*	Forward Reverse	5′-CCACTGAACCAAAAAGAAATCCC-3′ 5′-GAAAACAACACATAGCCAAACGC-3′
*COL I*	Forward Reverse	5′-CGATGGATTCCAGTTCGAGTATG-3′ 5′-TGTTCTTGCAGTGGTAGGTGATG-3′
*β*-*Catenin*	Forward Reverse	5′-AAGTTCTTGGCTATTACGACA-3′ 5′-ACAGCACCTTCAGCACTCT-3′
*GAPDH*	Forward Reverse	5′-TCTCCTCTGACTTCAACAGCGACA-3′ 5′-CCCTGTTGCTGTAGCCAAATTCGT-3′

## Data Availability

The data used to support the findings of this study are available from the corresponding authors upon request.
